# Immune checkpoint inhibition and heart injury: molecular mechanisms and clinical challenges

**DOI:** 10.1093/eschf/xvaf015

**Published:** 2026-01-13

**Authors:** Shan Lv, Jianan Xu, Liyue Yang, Junfeng Cui, Yuning Xin, Yan Zhao, Pengfei Li, Huize Han, Jian Li, Aidong Liu

**Affiliations:** Department of Cardiology, The Affiliated Hospital to Changchun University of Chinese Medicine, Changchun 130021, China; Department of Pulmonary Oncology, The Affiliated Hospital to Changchun University of Chinese Medicine, Changchun 130021, China; Department of Neurology, Changchun Hospital of Traditional Chinese Medicine, Changchun 130051, China; Department of Cardiology, The Affiliated Hospital to Changchun University of Chinese Medicine, Changchun 130021, China; Department of Cardiology, The Affiliated Hospital to Changchun University of Chinese Medicine, Changchun 130021, China; Department of Pulmonary Oncology, The Affiliated Hospital to Changchun University of Chinese Medicine, Changchun 130021, China; Department of Nephrology, The Affiliated Hospital to Changchun University of Chinese Medicine, Changchun 130021, China; College of Traditional Chinese Medicine, Changchun University of Chinese Medicine, Changchun 130117, China; College of Traditional Chinese Medicine, Changchun University of Chinese Medicine, Changchun 130117, China; Department of Cardiology, The Third Affiliated Clinical Hospital to Changchun University of Chinese Medicine, No. 1643, Jingyue Street, Changchun, Jilin Province 130022, China

**Keywords:** Immune checkpoint inhibitors, Targeted therapies, Immunotherapy, Cardiac injury, Drug-induced cardiomyopathy

## Abstract

Immune checkpoint inhibitors (ICIs), such as PD-1, PD-L1, and CTLA-4 inhibitors, enhance immune function by targeting T-cell surface receptors, overcoming natural immune inhibition. These drugs activate the immune system to detect and target cancer cells, providing an alternative therapeutic approach, particularly in cases where conventional treatments fail. However, the growing use of ICIs has highlighted several defence-related adverse events, particularly cardiac toxicity, including acute myocarditis, atherosclerosis, and heart failure unrelated to myocarditis. These complications present significant challenges, limiting the broader use of ICIs. Research into the cardiac toxicity of ICIs is still in its early stages, and the underlying mechanisms remain unclear. This review consolidates current findings on ICI-associated heart damage, explores potential factors contributing to ICI-induced cardiac injury, and evaluates preventive and therapeutic strategies. This work aims to provide a theoretical foundation for medical intervention and prevention of ICI-related cardiac damage.

## Graphical Abstract

**Graphical Abstract:**
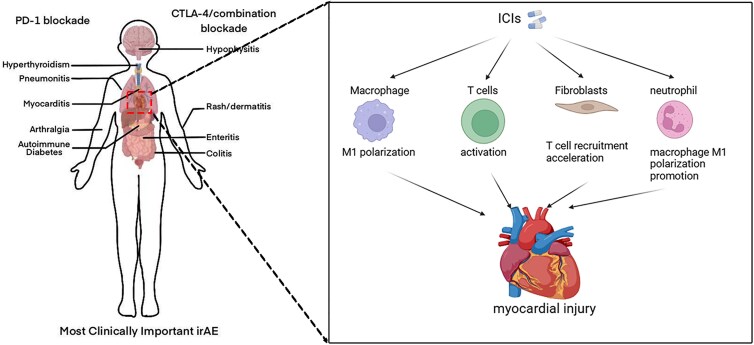
The figure briefly illustrates the mechanism of myocardial injury induced by ICIs

## Introduction

Biological regulators consist of molecules located across cell membrane, primarily formed by cytotoxic T lymphocyte antigen 4 (CTLA-4) as well as programmed cell death protein 1 (PD-1), both crucial for controlling onset, persistence, as well as strength in the body’s protective reactions.^1,2^ The immune synapse is a specialized contact area formed between immune cells, primarily occurring between T cells and antigen-presenting cells. In this region, T cells transmit signals to antigen-presenting cells through receptor-ligand interactions, thereby initiating the immune response and precisely regulating the onset and duration of the immune reaction.^1,2^ Specifically, CTLA-4 restrains T lymphocyte stimulation at an early stage by binding to ligands from the B7 family, such as B7-1 as well as B7-2.^3^ Alternatively, PD-1 suppresses functional activities in T cells when it attaches with PD-L1, a ligand predominantly found in cancerous tissue as well as certain normal tissues. This engagement is particularly relevant within a cancerous milieu, where triggering PD-1/PD-L1 signalling cascade enables tumour cells in evading the host’s defence mechanisms.^4^ The strategy for immune escape enables cancerous growths to escape the host’s protective response recognition, promoting their growth and spread to other sites in the body.^5,6^

As a result, immune checkpoint inhibitor (ICI), comprising anti-CTLA-4 as well as anti-PD-1 agents, function by removing such suppressive effects, thereby activating T cells and boosting the body’s defence capacity for detecting as well as eliminate cancerous growths. Such agents serve a crucial function in cancer immunotherapy.^7,8^ Immune checkpoint inhibitors have become especially important in managing advanced and metastatic malignancies. Such therapies counteract immune suppression, stimulating T lymphocytes for targeting and destroying tumour tissue, ultimately resulting in anti-tumour effects. Immune checkpoint inhibitors exhibit significant therapeutic impact in various malignancies, such as skin tumours, advanced pulmonary carcinoma, kidney malignancy, upper respiratory tract neoplasms, urinary tract malignancy, as well as Hodgkin’s disease.^9–14^ Immune checkpoint inhibitors’ application has provided new hope for patients, with some achieving long-term remission and sustaining progression-free survival even after the completion of treatment.

**Figure 1:**
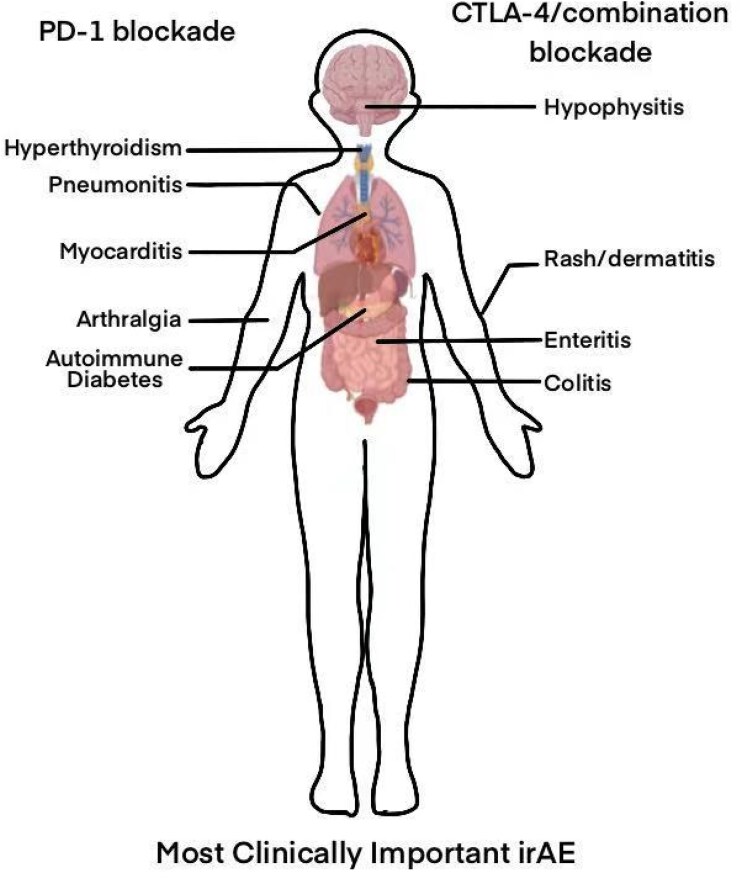
Major side effects induced by immune checkpoint inhibitors. Immune checkpoint inhibitors can cause various adverse reactions such as hyperthyroidism, pneumonitis, myocarditis, and arthralgia. CTLA-4, cytotoxic T lymphocyte antigen 4; irAE, immune-related adverse events; PD-1, programmed cell death protein 1

Immune checkpoint inhibitors may lead to immune-mediated effects on healthy tissues or organs. Although ICIs exhibit substantial efficacy for cancer therapy, they may give rise to several immune-related adverse events (irAEs), including skin reactions (such as rashes and itching),^15,16^ gastrointestinal issues (such as diarrhoea and abdominal pain),^17,18^ endocrine disorders (such as thyroiditis and adrenalitis),^19^ pulmonary complications (such as immune pneumonia),^20,21^ liver conditions (such as immune hepatitis),^22^ neurological effects (such as neuritis and myasthenia),^23,24^ and renal issues (such as immune nephritis).^25,26^ Among these, cardiac injury resulting from ICIs is relatively common and may lead to mortality in about 50% of patients^27,28^ (*Figure 1*), severely restricting the use of these medications in cancer treatment. Furthermore, there are currently no targeted treatments available for myocardial damage caused by ICIs, and only symptomatic supportive care can be provided once ICIs are discontinued.^29,30^ This challenge arises primarily due to the insufficient comprehension regarding processes responsible for ICI-related cardiac injury. To effectively address this issue, it is crucial to intensify research into the mechanisms behind myocardial injury triggered by immune checkpoint inhibitors. A comprehensive investigation into how the immune system attacks heart tissue under the influence of ICIs, alongside uncovering biochemical processes involved in immune-driven myocarditis as well as cardiac injury, will establish a foundation to create more targeted therapeutic approaches.

## Immune checkpoints and immune checkpoint inhibitors

Immune checkpoints are molecules that regulate immune responses, mainly by suppressing the overactivation of the immune system, thus maintaining immune tolerance and preventing autoimmune reactions. Common examples of immune checkpoint molecules include PD-1, CTLA-4, lymphocyte-activation gene 3, and T-cell immunoglobulin and mucin-domain containing-3 (TIM-3).^31^ PD-1 is an inhibitory receptor found by various elements within body immune system, such as T cells, B cells, NK lymphocytes, as well as dendritic cells.^32^ When PD-1 binds to its ligand PD-L1, it suppresses T lymphocyte stimulation as well as proliferation, particularly within neoplastic milieu, where neoplastic cells express PD-L1, thereby escaping body defence system recognition^33^

In addition to the PD-1/PD-L1 interaction, CTLA-4 also performs a crucial function within regulating the body’s defence mechanisms. This molecule is expressed on T lymphocyte surfaces, preventing impedes T lymphocyte stimulation through interaction with B7-1/B7-2.^34^ Despite both PD-1 and CTLA-4 playing inhibitory roles in immune responses, their functions in regulating T cells differ. Specifically, PD-1 mainly suppresses T lymphocyte effector activities via engagement by PD-L1, particularly within the tumour microenvironment. The PD-1 exerts negative feedback regulation during the later stages in T lymphocyte stimulation, primarily by inhibiting T lymphocyte proliferation as well as cytokine secretion, thus limiting sustained T lymphocyte activity. The mechanism helps prevent the body'’ defence response, ensuring it does not become overactive, thus protecting normal tissues from damage.^35–37^

**Figure 2:**
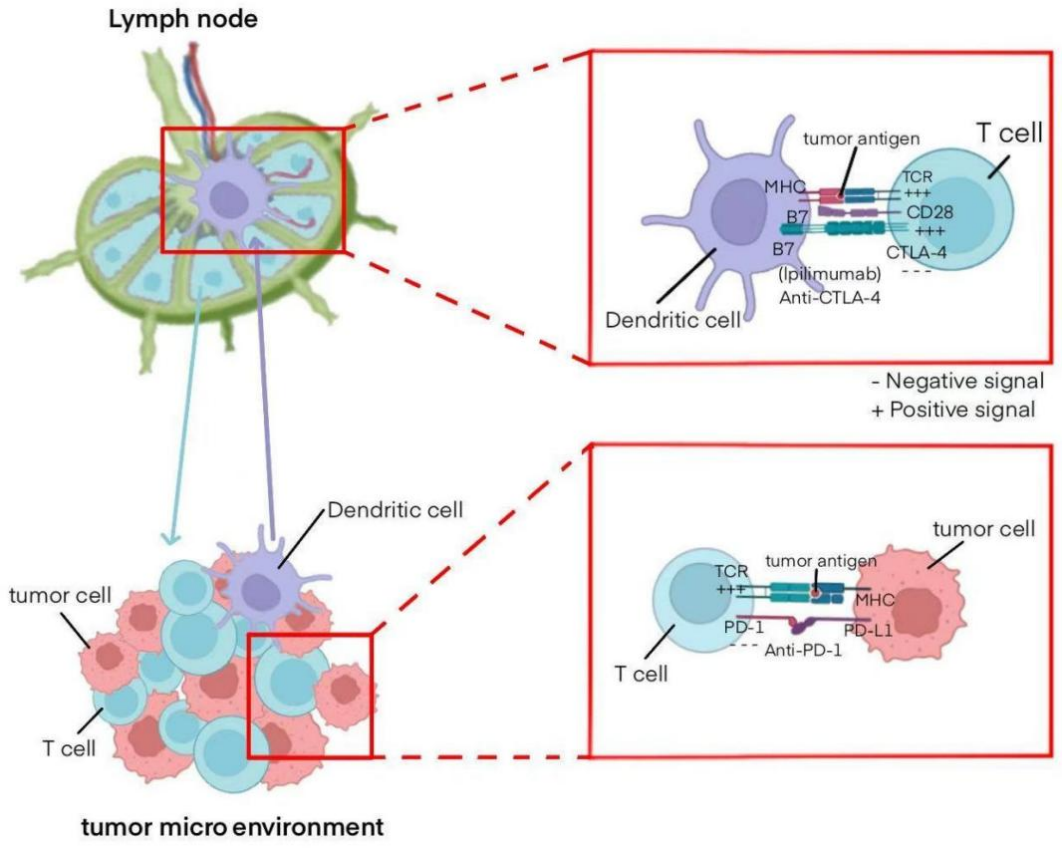
Process in immunological control point regulation. T-cell stimulation depends on presentation of antigens (e.g. cancer-related proteins) to T-cell receptors through MHC, along with secondary signals, such as interaction between cluster of differentiation 28 and B7. Additionally, T lymphocytes display cytotoxic T lymphocyte-associated protein 4; upon interaction to B7, delivers inhibitory signals that inactivate the T cells. Monoclonal agents directed against cytotoxic T lymphocyte-associated protein 4, like pembrolizumab, inhibit interaction of cytotoxic T lymphocyte-associated protein 4 to B7, which facilitates T lymphocyte stimulation while amplifying host defence activity targeting the presented antigen (i.e. cancer). Programmed cell death protein-1 is expressed by stimulated T lymphocytes, and its binding with programmed cell death ligand-1, found at neoplastic tissues, results in diminished T lymphocyte function. Through suppression, such binding is inhibited, anti-programmed cell death protein-1 tumour immunotherapy agents to promote T-cell activation to combat cancer. B7, a family of co-stimulatory molecules (B7-1 and B7-2); CD28, cluster of differentiation 28; CTLA-4, cytotoxic T lymphocyte-associated protein 4; MHC, major histocompatibility complex; PD-1, programmed cell death protein 1; PD-L1, programmed cell death ligand 1; TCR, T cell receptor

In contrast, CTLA-4 primarily functions in the initial stage within T lymphocyte stimulation. Its main function is to suppress T lymphocyte stimulation through interacting with B7-1/B7-2. CTLA-4 - defined earlier in the text vies against CD28 present at T lymphocytes surfaces to engage with B7 molecules, which typically provide immune-stimulatory signals through the association of CD28, consequently promoting T lymphocyte stimulation.^38–40^ By competing for binding, CTLA-4 reduces the activation signals for T cells, limiting their initial response to antigens. This effect predominantly occurs when T lymphocytes first engage with pathogen-presentation agents (for instance, immune-related structures), which is why CTLA-4 primarily regulates the early stage of immune responses (*Figure 2*).

Lymphocyte-activation gene 3 as well as TIM-3 represent additional key checkpoint molecules present in T lymphocytes and NK lymphocytes, serving a crucial function within negative regulation of physiological responses.^41–43^ These immune checkpoint molecules interact with their respective ligands, leading to reduced T cell activity, which hinders the immune system's ability to target tumour cells and helps tumour cells evade immune surveillance.

Immune checkpoint inhibitors represent categories in drugs which restore T lymphocyte function by overcoming the suppression imposed by immune checkpoints, thus enhancing the immune system’s capacity to target and eliminate tumours. Notable immune checkpoint inhibitors include anti-PD-1 antibodies, such as nivolumab and pembrolizumab, and anti-CTLA-4 antibodies, such as ipilimumab, as listed in *Table 1*. These drugs work by inhibiting interactions involving cellular regulatory receptors as well as specific molecular partners, which alleviates suppression of T cells and strengthens T lymphocyte function, thus amplifying body defence mechanisms targeting malignancies. For example, anti-PD-1 agents promote T lymphocyte recognition as well as destruction in neoplastic tissues by preventing PD-1 from interacting with PD-L1, while anti-CTLA-4 inhibitors improve T-cell activity against tumours by lifting the suppression that CTLA-4 exerts over T lymphocytes. These agents demonstrate considerable effectiveness at managing multiple types of malignancy, such as skin cancer, non-small cell lung cancer, renal cell carcinoma, as well as others.^60–64^ Immune checkpoint inhibitors improve the cancer-fighting capabilities of body defence mechanisms, resulting in prolonged remission in some cancer patients. However, these drugs can also induce autoimmunity-associated complications, for example, rashes, liver dysfunction, as well as enteritis (*Table 1*), necessitating close monitoring and management during clinical use.

**Table 1:** Commonly used immune checkpoint inhibitors in clinical practice and their indications for various cancers

Drug name	Type	FDA approval year	FDA-approved cancer	Side effects	References
**Ipilimumab**	CTLA-4	2011	Melanoma, kidney neoplasm, intestinal malignancy	Myocarditis, arrhythmias, pericarditis, rash, fatigue, decreased appetite, autoimmune-associated complications, for instance, colitis, liver dysfunction, endocrine disorders, etc.	^44–46^
**Nivolumab**	PD-1	2014	Melanoma, non-small cell lung cancer, renal cell carcinoma, head and neck epithelial tumour, bladder tumour, colon tumour, liver tumour	Myocarditis, arrhythmias. pericarditis, rash, fatigue, pneumonia, hepatitis, colitis, renal dysfunction, etc.	^47–49^
**Pembrolizumab**	PD-1	2014	Melanoma, NSCLC, SCLC, primary CNS tumour, aggressive B-cell malignancy, urothelial cancer, stomach malignancy, oesophageal malignancy, uterine malignancy, liver malignancy, Merkel neoplasm	Myocarditis, arrhythmias. pericarditis, fatigue, rash, colitis, pneumonia, hepatitis, kidney injury, etc.	^50,51^
**Cemiplimab**	PD-1	2018	Cutaneous squamous cell carcinoma	Myocarditis, arrhythmias, pericarditis, rash, inflammation of the colon, liver inflammation, pneumonia, etc.	^52,53^
**Avelumab**	PD-L1	2017	Merkel cell carcinoma	rash, liver damage, immune pneumonia, etc.	^54^
**Atezolizumab**	PD-L1	2016	Urothelial carcinoma, NSCLC	pneumonia, colitis, rash, liver damage, etc.	^55,56^
**Durvalumab**	PD-L1	2017	Urothelial carcinoma	Myocarditis, arrhythmias, pericarditis dermatological reactions, liver damage, gastrointestinal inflammation, etc.	^57–59^

B-cell, B lymphocyte (type of white blood cell); CNS, central nervous system; Urothelial carcinoma, a cancer that originates in the urothelium, the tissue lining the urinary tract; CTLA-4, cytotoxic T lymphocyte-associated protein 4; FDA, food and drug administration; NSCLC, non-small cell lung cancer; SCLC, small cell lung cancer; PD-1, programmed cell death protein 1; PD-L1, programmed cell death ligand 1; Merkel cell carcinoma, a rare and aggressive skin cancer.

## Toxic impact caused by immune checkpoint inhibitors to cardiac function: epidemiology, clinical features, diagnosis, and management

Following the authorization of ipilimumab, a pioneering CTLA-4 blocker for advanced skin cancer in 2011, several ICIs subsequently received approval to across numerous malignancies, such as pulmonary carcinoma, genitourinary cancers, head and neck cancers, gastrointestinal cancers, breast cancer, and gynaecological cancers. The number of patients receiving ICIs has been steadily increasing. By 2018, 14% of patients in the U.S. had received ICIs, and this percentage rapidly rose to 22% by 2020.^65^ As the administration of ICIs grows, attention to their immune-related side effects has also intensified.

Although myocardial injury associated with ICIs occurs at a significantly lower rate than skin toxicity (72%) and gastrointestinal toxicity (8%–27%),^66^ the first documented case of myocardial injury linked to ICIs was published in 2016.^67^ Meta-analyses of RCT as well as pharmacovigilance data show incidence in ICI-induced myocardial injury remains relatively low, ranging from 0.27% to 0.67%. However, this rate is higher, at 1.3%, in patients undergoing combination ICI therapy.^68^ A 2016 multicentre registry standardized case identification through clinical criteria and biopsy, reporting an incidence of 1.14% for myocardial injury caused by ICIs.^28^ Furthermore, prospective monitoring studies have documented a higher range of incidence, from 1.40% to 2.46%.^69^

Despite its lower frequency, fatality incidence associated with ICI-associated myocardial injury remains notably elevated, fluctuating across a range extending from 30% to 50%. Such circumstances emphasize a vital need for prompt identification as well as management, given the growing population undergoing ICI interventions coupled with a severe prognosis associated with ICI-induced myocardial injury.^70^

Myocardial injury resulting from ICIs typically manifests over a period spanning from several weeks to a few months following the commencement in therapy. Nevertheless, several reports indicate that symptoms may emerge even years later. The clinical presentation of this condition often includes breathlessness, irregular heartbeats, discomfort in the thoracic region, extreme tiredness, fainting, and symptoms associated with cardiac insufficiency.^71–73^ In certain cases, patients may experience acute cardiac failure, conduction abnormalities, or persistent myocardial dysrhythmias. On the other hand, some patients remain asymptomatic and are only identified through biomarkers or abnormal findings in cardiac imaging.^74,75^ Furthermore, evidence suggests that ICI-induced myocardial injury may co-occur with additional irAEs, including muscle inflammation and neuromuscular weakness, which present with symptoms like muscle pain, weakness, diplopia, and dysphagia. These patients face an increased risk of respiratory failure, cardiogenic shock, life-threatening arrhythmias, and death.^76,77^

Diagnosing myocardial injury induced by ICIs is a challenging task and primarily relies on clinical symptoms, cardiac biomarkers (including cardiac enzymes), electrocardiogram (ECG), advanced diagnostic methods (such as ultrasound-based cardiac assessment or MRI of the myocardium), and tissue pathology.^78,79^ Elevated levels of troponin are essential for diagnosis, with troponin T showing greater sensitivity than troponin I in myocardial injury caused by ICIs cases. Cardiac magnetic resonance has been considered among the most dependable imaging tool in assessing myocardial injury caused by ICIs, providing valuable imaging evidence of cellular oedema as well as non-oxygen-related damage in cardiac tissue. Although myocardial biopsy remains the definitive diagnostic standard, its clinical application is constrained because of the procedural invasiveness and potential sampling errors.^80,81^

The main treatment approach involves the administration of high-dose corticosteroids (e.g. 500–1000 mg daily) to regulate the immune response. Initiating corticosteroid therapy early has been shown to decrease the occurrence of cardiac events, particularly when treatment is started within 24 h of the initial symptoms.^82,83^ In cases where individuals fail to show an adequate improvement with corticosteroids or exhibit persistently elevated cardiac biomarkers, administering immune-modulating agents such as cyclosporine, anti-thymocyte globulin, or monoclonal antibody therapies may be considered as part of a combined treatment strategy.^30,84–86^ Although current therapies can improve patient prognosis to some extent, the therapeutic outcomes for myocardial injury caused by ICIs remain suboptimal, and there is a lack of targeted treatments and standardized treatment guidelines within healthcare settings. This is largely attributed to incomplete understanding regarding the factors underlying ICI-induced myocardial injury, necessitating further basic research to clarify these mechanisms.

## Mechanisms of immune checkpoint inhibitors-induced myocardial injury

Immune checkpoint inhibitors amplify immune response in T lymphocytes through blockade at self-regulatory sites. While this activation is beneficial for targeting tumour cells, it can also trigger the immune system to attack normal cardiac tissue. When T cells become excessively active, they may target myocardial cells, resulting in myocarditis and other types of cardiac damage. As a result, numerous studies have suggested that T cells are central to the development of ICI-induced myocardial injury.^87–89^ For example, Tay WT’s research demonstrated that applying ICIs to myocardial cells alone does not induce apoptosis. However, when myocardial cells are co-cultured with T cells and exposed to ICIs, myocardial damage occurs.^90^ Further advancing this work, Axelrod ML *et al*.^91^ have shown how T lymphocytes targeting α-actin contribute significantly to the heart muscle inflammation induced by immune checkpoint inhibitors.

Ma P *et al*.^92^ identified how inflammatory mediators are essential to progression associated with the condition. Specifically, the team utilized individual cell transcriptome profiling within a rodent system for ICI-induced myocardial injury and observed a significant increase in C-C Motif Chemokine Receptor 2 (CCR2) myeloid-associated phagocytes as well as CD8 T lymphocytes. Phagocyte populations exhibited considerable heterogeneity, with inflammatory CCR2 subsets derived from CCR2 monocytes showing marked enrichment. These subsets expressed high levels of C-X-C motif chemokine ligand 9 (Cxcl9), C-X-C motif chemokine ligand 10 (Cxcl10), guanylate binding protein 2B (Gbp2b), and Fc gamma receptor 4 (Fcgr4). Notably, in patients diagnosed with ICI-induced myocarditis, macrophage populations positive for CXCL9, CXCL10, as well as CD16α (the human counterpart to murine FcgR4) showed significant expansion. Computational analyses of intercellular interactions suggest that T cells engage with Cxcl9Cxcl10-expressing phagocytes through the interferon gamma (IFN-γ) as well as CXCR3 receptor activation. The depletion of CD8 T lymphocytes or myeloid-derived populations, combined with inhibition of IFN-γ communication, resulted in a reduction in proliferation among Cxcl9Cxcl10-expressing inflammatory populations within cardiac tissue, ultimately alleviating inflammation in the myocardial tissue, supporting the idea whereby a specific relationship is central to development associated with such a condition. Interestingly, the team extended their research and found that inhibition of CXCR3 notably increased recovery prospects in rodents suffering from ICI-induced myocardial injury.^93^ Their sequential experiments revealed the critical role of macrophages in ICI-induced myocardial injury, demonstrating that this injury depends on specific macrophages expressing molecules that recruit T cells to the myocardium, thereby facilitating damage. However, it has yet to be experimentally validated whether macrophages themselves directly contribute to the injury, which remains a limitation of the team’s findings. Similarly, Cao *et al*. also focused on macrophages and found that ICIs directly influence these cells, inducing M1 polarization and resulting in myocardial inflammation and damage^94^ focused on macrophages and found that ICIs directly influence these cells, inducing M1 polarization and resulting in myocardial inflammation and damage^92^ and Chen Y^95^ reported comparable results, observing that ICIs promote M1 polarization, though through distinct signalling pathways.^96^ However, although basic experiments have shown that inhibiting macrophage polarization, especially M1 polarization, can effectively alleviate ICI-induced myocardial injury, for tumour tissues, M1 macrophages can suppress tumour growth. Therefore, simply inhibiting M1 polarization of macrophages in the body may alleviate ICI-induced myocardial injury, but from a theoretical perspective, it could also weaken the anti-cancer effects of ICIs. This issue could be addressed by constructing specific materials, such as nanoparticles targeted at macrophages in myocardial tissue.

In contrast to Ma P’s team, Cao^94^ and Hou *et al*. contend that ICIs induce myocardial damage by directly influencing macrophage polarization, without involving T cells in the process. However, a significant issue with this conclusion arises: from a mechanistic perspective, ICIs predominantly target T cells. Thus, the question remains, how do they impact macrophages? A similar issue was raised in Xia W’s team’s study,^97^ which also examined the effects of ICIs on macrophages. Their findings diverged from those of Cao ’s team, showing that ICIs induce macrophages to secrete exosomes, which contribute to myocardial cell senescence. Apart from macrophages, myocardial fibroblasts have been implicated with mobilizing T lymphocytes into myocardium.^98^ Studies have shown that in mice with ICI-induced myocardial injury, angiopoietin-like protein 2 (ANGPTL2) expression is increased during ICI-associated cardiac inflammation. ANGPTL2 absence significantly alleviates inflammation-driven tissue damage within the mice, corresponding to reductions in T cell as well as macrophage numbers. Mechanistically, ANGPTL2 produced by myocardial fibroblasts stimulates cytokines production through NF-κB molecular cascade, promoting T lymphocyte infiltration into cardiac tissue and thereby exacerbating ICI-induced myocardial injury.^98^ Wu *et al*.^99^ also observed that neutrophils play a role in this process. Their study demonstrated that anti-CTLA-4 m2a antibodies increase Cxcl1 produced by cardiac fibroblasts (CF), facilitating neutrophil infiltration into the myocardial zone in EAM mice through elevated Cxcl1-Cxcr2 migration. Moreover, the Ccl5-neutrophil subset along with related pro-inflammatory molecules/mediators promote microphage (Mϕ) activation towards the M1 phenotype. Such immune changes alter the pattern of immune cell infiltration and phenotypic shifts, while pro-inflammatory factors further exacerbate cardiac injury. This suggests that the Ccl5-neutrophil subset plays a crucial role in exacerbating cardiac injury induced by anti-CTLA-4 m2a antibodies in EAM mice. Zhang *et al*. further discovered that PD-1 inhibitors do not exert an immediate influence over CF, but instead modulate their activity through endothelial cell-cardiac fibroblast interaction. The PD-1 inhibitors reduce TGF-β1 production within endothelial cells through suppression in TCF12, considered the gene regulatory factor for TGF-β1. The reduction in TGF-β1 secretion consequently lowers CF function, resulting in a reduction in extracellular matrix accumulation. Furthermore, PD-1 inhibitors induce endothelial-mesenchymal transition (EndMT), promoting surrounding tissue fibrosis. Vascular impairment resulting from PD-1 inhibitors results from ROS build-up within ECs. Blocking ROS using NAC reversed TCF12 downregulation in ECs and EndMT, thereby preserving proper extracellular matrix organization as well as heart performance among PD-1 antagonist-administered mice.^100^

The research conducted by Sun *et al*. is particularly significant, as they revealed that ICI-induced myocardial injury leads to an increase in pyroptosis in both human and mouse hearts. Moreover, they demonstrated that targeting pyroptosis could effectively mitigate this injury.^101^ This pyroptosis is primarily initiated by the myocardial cells themselves, rather than immune populations, contributing significantly to this process. In a similar vein, Wu *et al*.^102^ also identified that pyroptosis contributes significantly to myocardial injury caused by the combined impact of ICIs and radiation. Both research teams have validated this novel mechanism through extensive experimentation. However, these findings raise several critical questions. For instance, given that ICIs are immune checkpoint inhibitors designed to block immune checkpoint interactions, how does this mechanism affect myocardial cells and increase their pyroptosis? Is this effect the result of direct action on myocardial cells, or does it indirectly promote pyroptosis in myocardial cells through immune cells? Furthermore, Within the framework ICI-related heart damage, do the infiltrating immune cells, as previously proposed by other studies, result from ICI’s direct effect on immune cells, or do they arise due to ICI-induced myocardial pyroptosis regulating immune cell infiltration? These questions could potentially be addressed through more comprehensive cell-based experiments. However, the lack of *in vitro* validation in the experimental design significantly undermines the reliability of these conclusions.

## New strategies for preventing and treating immune checkpoint inhibitor-induced cardiac injury

Corticosteroid therapy is still the standard treatment for ICI-induced myocardial injury in clinical practice, a strategy based on the prior understanding that T cells predominantly mediate the pathogenesis of this injury. In alignment with this mechanism, several studies have focused on inhibiting immune cell activation to mitigate the effects of ICI-induced myocardial injury. For instance, in preclinical studies, Michel’s team^103^ demonstrated that blocking TNF-α, a pivotal inflammatory mediator significantly influences the stimulation of immune defences as well as inflammation, particularly in cardiac injury, could provide therapeutic benefit. Their findings indicated that inhibiting TNF-α could effectively reduce ICI-induced myocardial injury. However, they did not provide detailed insights into the mechanism by which TNF-α functions as a drug target.

Recent advances in deciphering the pathophysiology of cardiotoxicity associated with ICI, new therapeutic strategies have been validated in preclinical settings. For example, Ma P’s team discovered that macrophages and T cells interact through the IFN-γ as well as CXCR3 transduction mechanisms to influence myocardial injury in ICI-induced myocardial damage. Their experiments demonstrated that targeting CXCR3 could effectively reduce myocardial injury, indicating that developing drugs aimed at CXCR3 holds promise as a novel therapeutic approach.^93^

Moreover, both Ma’s team^93^ and Cao’s team^94^ found that inhibiting macrophage activity could alleviate ICI-induced myocardial injury. Cao’s team further identified that inhibitors targeting the macrophage polarization pathway (cGAS/STING pathway) could significantly decrease the severity of myocardial injury in ICI-treated mice.

In addition, Sun’s team explored the role of myocardial cell pyroptosis in ICI-induced myocardial injury and provided evidence that targeting pyroptosis could effectively reduce myocardial injury, offering a potential new treatment option for patients.^101^ Although studies by Gergely^104^ and Dimitriou^105^ did not fully elucidate the specific mechanisms behind ICI-induced myocardial injury, they suggested that drugs inhibiting IL-17A (a cytokine) could be beneficial in preventing and treating ICI-induced cardiac dysfunction.

Contrasting with the role of myocardial pyroptosis, Sun’s team found that inflammatory cells primarily contribute to myocardial damage by promoting necroptosis in myocardial cells. Their findings showed that inhibiting necroptotic cell death could effectively mitigate ICI-induced myocardial injury. Since their research did not negate the involvement of inflammatory cells in this process, it instead demonstrated that once inflammatory cells infiltrate the myocardium, they predominantly induce necroptosis in myocardial cells, which leads to myocardial injury. This conclusion, therefore, appears more logically consistent.^106^

Studies have demonstrated that combining periodic fasting-mimicking diet (FMD) cycles with anti-PD-L1 therapy proves more effective in slowing melanoma progression in mice compared to the use of immune checkpoint inhibitors alone. Furthermore, this combined approach significantly alleviates cardiac fibrosis, necrosis, and hypertrophy induced by immune checkpoint inhibitors. It also reduces the infiltration of CD3 and CD8 T cells into myocardial tissue and diminishes both systemic and myocardial markers of oxidative stress and inflammation.^107^ While the feasibility of this dietary strategy remains uncertain, pharmacological treatments with comparable mechanisms are easier to implement.^108^

In their research, Huang’s team found that the immunomodulatory drug leflunomide can alleviate ICI-induced myocardial injury. Further mechanistic investigations revealed that leflunomide does not operate by modulating immune responses but by modifying the gut microbiota, which leads to the production of indole-3-propionic acid that protects the heart.^108^ Building on these findings, we hypothesize that periodic FMD cycles may also protect the myocardium through similar modulation of the gut microbiota. However, pharmacological treatments appear to provide a more cost-effective solution with similar protective benefits, positioning them as a promising therapeutic alternative.

Several studies have explored various compounds and natural ingredients. For example, research conducted by He *et al*. showed that patients with ICI-induced myocardial injury exhibited elevated serum levels of L-kynurenine, a product through IDO1, compared to those presenting non-ICI-induced myocardial injury. Both cell culture systems and animal models evidence suggest omitted L-KYN may offer clinical benefits for treating ICI-induced acute myocarditis through suppressing leukocyte differentiation towards anti-pathogenic phenotypes as well as inhibiting production of pro-pathogenic signalling molecules. At the molecular level, L-KYN improves myocardial performance primarily through interfering with JAK1/STAT3 signal transduction.^109^ These findings propose that exogenous supplementation with L-kynurenine could emerge as a potential therapeutic strategy against ICI-induced cardiac damage in the future. Furthermore, Zhang’s team discovered that saffron extract treatment could enhance heart function, alleviate myocardial inflammation and fibrosis, and partially reverse ICI-associated myocarditis. Mechanistically, ICI treatment activates the NLRP3 inflammasome in myocardial cells expressing NLRP3. Saffron extract significantly downregulates NLRP3, activated GSDMD, processed caspase-1, IL-1β, along with IL-18.^110^

Exosomes represent nanometre-scale extracellular particles (30–150 nm) actively released from cellular sources, contain diverse biomolecular constituents, including enzymes, fats, as well as RNA.^111^ These exosomes are crucial for mediating cell-to-cell signalling, facilitating material exchange, and modulating immune responses.^112^ Lately, the focus on utilizing exosomes in therapeutic applications across different diseases. In the case of ICI-induced myocardial injury, Zhou’s team showed that extracellular vesicles isolated from hBMSC-Exos help mitigate myocardial injury induced by ICIs, primarily through modulating M1 phagocyte activation, as well as influencing cardiac inflammatory apoptosis.^113^

## Conclusion

**Figure 3:**
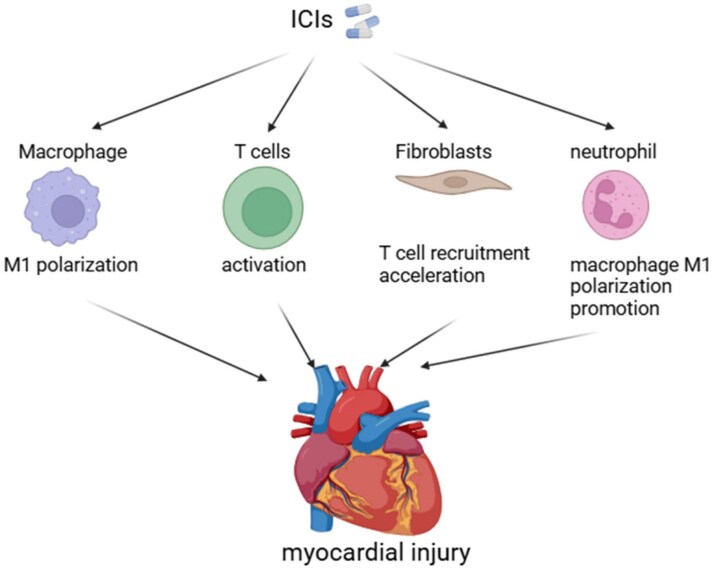
The mechanism of myocardial injury caused by immune checkpoint inhibitors. Immune checkpoint inhibitors lead to myocardial injury by promoting macrophage M1 polarization, activating T cells, or facilitating T-cell recruitment to the heart through fibroblasts, or by promoting macrophage M1 polarization via neutrophils

Immune checkpoint inhibitors function by reversing immune checkpoint inhibition, thus boosting a body’s defence mechanism’s capacity for identifying and attacking tumours. This mechanism has introduced novel therapeutic possibilities aimed at individuals suffering from various forms of malignancy. However, as clinical application of ICIs expands, the risk for system-mediated side effects, especially cardiovascular injury, has become more evident, posing significant challenges in the management of patients. While the incidence of myocardial injury associated with ICIs remains relatively low, Fatalities linked to the illness remain strikingly elevated, ranging from 30% to 50%. Exact processes underlying ICI-driven myocardial injury remain inadequately explored, but research indicates that intricate interactions between T cells, macrophages, fibroblasts, and myocardial fibres could be crucial to this process (*Figure 3*). Specifically, ICIs may worsen cardiac inflammation as well as damage by inducing M1 macrophage polarization, even though underlying processes through how ICIs affect macrophages are not fully understood. Moreover, myocardial fibroblasts may contribute to myocardial injury through their role in T cell recruitment and collagen deposition. Alternatively, some studies suggest that ICIs could cause direct damage to myocardial cells, such as through the activation of myocardial pyroptosis. Nonetheless, this theory seems to conflict with the established pharmacological actions of ICIs and is not supported by adequate cellular-based experimental validation, which raises concerns regarding its reliability.

While intensive steroids remain the main therapy in ICI-induced myocardial injury, their limited effectiveness and the lack of standardized protocols emphasize the urgent need to develop novel therapeutic approaches. Recent studies on the mechanisms of ICI-induced myocardial injury have uncovered significant advances, providing new perspectives on the roles of macrophage polarization, T-cell activation, and myocardial pyroptosis, each of which presents a potential target for therapeutic interventions. Drugs designed to target CXCR3, pyroptosis, and necroptosis offer the possibility of opening new treatment avenues. Moreover, research has shown that periodic fasting-mimicking diets can significantly alleviate myocardial injury induced by ICIs, likely through their effects on the gut microbiota. In parallel, the immunomodulatory drug leflunomide has been found to alleviate ICI-induced myocardial injury by modulating the gut microbiota, thus expanding available clinical treatment options. While these findings are still in the early phases, they provide important theoretical insights and suggest practical avenues towards creating better therapeutic approaches ahead.

Finally, current research lacks a focus on the diagnosis of ICI-induced myocardial injury, particularly the development and validation of early biomarkers. This is crucial for patients with myocardial injury caused by ICIs and represents an important area for future research.

Cardiac injury caused due to ICIs represents multifaceted defence-mediated responses that involves the activation of various immune cell types. Continued exploration of the underlying mechanisms behind ICI-induced cardiac injury, along with advancing novel medical approaches, holds the capacity for significantly enhancing patient prognosis while optimizing clinical treatment effectiveness.
